# Comparisons of Ribosomal Protein Gene Promoters Indicate Superiority of Heterologous Regulatory Sequences for Expressing Transgenes in *Phytophthora infestans*

**DOI:** 10.1371/journal.pone.0145612

**Published:** 2015-12-30

**Authors:** Laetitia Poidevin, Kalina Andreeva, Careen Khachatoorian, Howard S. Judelson

**Affiliations:** Department of Plant Pathology and Microbiology, University of California, Riverside, California, United States of America; Agriculture and Agri-Food Canada, CANADA

## Abstract

Molecular genetics approaches in *Phytophthora* research can be hampered by the limited number of known constitutive promoters for expressing transgenes and the instability of transgene activity. We have therefore characterized genes encoding the cytoplasmic ribosomal proteins of *Phytophthora* and studied their suitability for expressing transgenes in *P*. *infestans*. *Phytophthora* spp. encode a standard complement of 79 cytoplasmic ribosomal proteins. Several genes are duplicated, and two appear to be pseudogenes. Half of the genes are expressed at similar levels during all stages of asexual development, and we discovered that the majority share a novel promoter motif named the PhRiboBox. This sequence is enriched in genes associated with transcription, translation, and DNA replication, including tRNA and rRNA biogenesis. Promoters from the three *P*. *infestans* genes encoding ribosomal proteins S9, L10, and L23 and their orthologs from *P*. *capsici* were tested for their ability to drive transgenes in stable transformants of *P*. *infestans*. Five of the six promoters yielded strong expression of a GUS reporter, but the stability of expression was higher using the *P*. *capsici* promoters. With the *RPS9* and *RPL10* promoters of *P*. *infestans*, about half of transformants stopped making GUS over two years of culture, while their *P*. *capsici* orthologs conferred stable expression. Since cross-talk between native and transgene loci may trigger gene silencing, we encourage the use of heterologous promoters in transformation studies.

## Introduction

Transformation-based technologies such as gene silencing, overexpression, and protein tagging have contributed much to our understanding of the biology of *Phytophthora*, a genus of oomycetes that includes many devastating plant pathogens. Important oomycetes include the potato late blight agent *P*. *infestans*, the soybean root rot pathogen *P*. *sojae*, and the broad host-range vegetable pathogen *P*. *capsici* [1]. Molecular genetic studies of *Phytophthora* spp. are becoming increasingly routine, but transgene expression can be unstable [2–4]. This challenge is shared with many other eukaryotes, especially plants [5] and animals [6].

Phenomena linked to transgene instability outside *Phytophthora* include DNA excision and, more often, epigenetic processes [5, 7]. The latter can result from insertions into existing heterochromatic regions that fail to support long-term gene expression, the assembly of heterochromatin on tandemly repeated transgene arrays [8], or homology-based silencing. Both transcriptional and post-transcriptional homology-based silencing may be caused by interactions between the construct and native DNA in the recipient, or between transgenes. When different genes utilizing the same promoter are stacked, *trans*-silencing may occur [9, 10]. This is not trivial to avoid, since it is often desirable to express two or more genes with similar patterns, and multiple functionally equivalent promoters may not be available. Resolutions to the problem could involve developing derivatives of a promoter in which its regulatory motifs are placed in novel context [11], or identifying new promoters.

Only a limited number of promoters have been used to express transgenes in *Phytophthora*. Most studies have employed the promoter from the *ham34* gene of the downy mildew *Bremia lactucae* [12–15]. Downy mildews form a sister clade to *Phytophthora* within the oomycetes [16]. An *hsp70* promoter from *B*. *lactucae* has also proved useful [17, 18]. Both promoters are believed to confer high, constitutive expression. Often a selectable marker is expressed from one promoter, and a gene of interest such as a fluorescently tagged protein from the other [19, 20]. In recent studies, we stacked as many as four transgenes in the same *P*. *infestans* strain [21]. This required the use of the same promoter more than once, which complicated cloning and raised concern about *trans*-silencing or recombination between the regulatory sequences. *Phytophthora* has typical eukaryotic pathways for gene silencing [22]. It also recombines transforming DNA molecules that contain homology with each other at high frequency [23], although homologous recombination between introduced DNA and chromosomal sequences is very infrequent [24].

Our main goal in the present study was to develop new constitutive promoters for expressing transgenes in *Phytophthora*, using regulatory sequences from genes encoding ribosomal proteins. Such promoters are typically strong [25] and have proved useful for expressing genes in a variety of systems [26–28]. We also sought to compare the effectiveness of homologous and heterologous promoters. Consequently, we identified genes for ribosomal proteins from the genome of *P*. *infestans* and *P*. *capsici*, studied their expression during the life cycle, and tested the activity of three of the promoters in stable transformants of *P*. *infestans*. Although a prior study reported the use of a L41 promoter from *Phytophthora sojae* [28], it was not tested here since the expression stability of its *P*. *infestans* orthologs during the life cycle was inferior to the three promoters selected for analysis. Promoters from genes encoding proteins S9 and L10 proved most useful in *P*. *infestans*. Interestingly, the most stable expression in *P*. *infestans* resulted from the use of the *P*. *capsici* promoters rather than their *P*. *infestans* orthologs.

## Materials and Methods

### Bioinformatics

*P*. *infestans*, *P*. *capsici*, and *P*. *parasitica* genes were extracted from fungidb.org [29] using searches for the Gene Ontology term "ribosome" (GO:0005840) and BLASTP queries using proteins from other species from the Ribosomal Protein Gene database [30]. When hits to conserved genes were not identified within the annotated gene sets, the *Phytophthora* genomes were searched by TBLASTN. Hits were validated by reciprocal best BLASTP. *P*. *infestans* and *P*. *capsici* gene numbers correspond to those in their respective databases at the Broad Institute (www.broadinstitute.org; v.2, strain T30-4) or Joint Genome Institute (genome.jgi.doe.gov; v.1, strain LT1534). MEME [31] was used to discover motifs as over-represented words in promoter datasets, using a series of searches of datasets containing 200 or 700-nt of DNA 5' of the start codon, and word sizes in the 5 to 10, 8 to 14, or 10 to 16-nt ranges. The number of hits described in Results are based on searching 700-nt of DNA 5' to each gene, using the FIMO search tool with a *p*-value cut-off of 10^−4^ [31]. The significance of association of a motif with a gene class was determined using Fisher's exact test. GO term enrichment analysis employed GoStat using the Benjamini correction method for false discovery [32]. Small nucleolar RNAs (snoRNAs) were identified using SnoGPS [33].

### Expression analysis

Microarray data were as described [34]. Array features corresponding to annotated genes were identified using BLASTN with a 97% identity cutoff. Expression stabilities of genes between different developmental stages were calculated based on their relative standard deviations from the mean expression level. RNA-seq data were obtained using RNA from paired-end libraries generated using the Truseq kit from Illumina. Reads were filtered for quality, adapters removed, aligned to the reference genome using Tophat, and FPKM (fragments per kilobase per million reads) values calculated. The RNA-seq data were also used to map transcription start sites. For quantitative reverse transcription-PCR (qRT-PCR) analysis of the *P*. *infestans* genes encoding RPL10 and RPS9, RNA was extracted using the Spectrum Plant kit (Sigma) from tissues ground under liquid nitrogen, treated with RQ1 DNAse (Promega), and cDNA synthesized using the Maxima RT-PCR kit (Thermo). After confirming that primer efficiencies were above 95%, amplifications were performed using the Dynamo SYBR Green kit (Thermo) with the following program: 95°C for 15 min, followed by 40 cycles of 94°C for 30 sec, 55°C to 60°C (depending on primer) for 30 sec, and 72°C for 30 sec. Melt curves were generated at the end of each run to test the fidelity of amplification. Expression levels were calculated using the ΔΔC_T_ method, using a gene PITG_11766 as a housekeeping control.

### Vector construction

Primers used for polymerase chain reaction (PCR) are provided in S1 Table. Promoters were amplified by PCR from isolates 1114 of *P*. *infestans* and LT1534 of *P*. *capsici*, respectively, and cloned as *Xba*I-*Eco*RI fragments into GUS reporter plasmid pNP-GUS [35]. Initially, primers were designed to amplify 500-nt fragments from the *P*. *infestans* and *P*. *capsici* promoters as listed in S1 Table. PCR-amplified portions of the promoter of *PcRPS9*, the *P*. *capsici gene* encoding protein S9, were also cloned into pTOR (Genbank accession EU257520.1) using *Sna*BI-*Eco*RI restriction sites. The GUS reporter was cloned into *Eco*RI-*Xba*I restriction sites downstream of the promoters.

### Manipulations of *P*. *infestans*

Transformations were performed as described using *P*. *infestans* strain 1306 [36]. Transformants were selected and maintained at 18°C in the dark on rye-sucrose agar containing 10 μg/ml G418. Expression of the β-glucuronidase (GUS) gene was measured initially using hyphae cut from colonies on the primary transformation plate. Positive clones then were subcultured and transferred to fresh plates every 30 days using plugs from the growing edge of each culture. Plant infections were performed by placing drops of zoospore suspensions on leaflets of tomato cultivar New Yorker.

### Reporter assays

Histochemical assays for GUS were performed as described using bromochloroindoyl-β-glucuronide [36], and scored after overnight incubation at 37°C. For *in planta* staining, infected leaflets were vacuum-infiltrated in staining solution, incubated overnight at 37°C, and then decolorized in ethanol. Quantitative assays for GUS were performed using about 300 mg mycelia from 7-day cultures, which were ground under liquid nitrogen, thawed in 300 μl of extraction buffer (50 mM NaHPO_4_ pH 7.0, 10 mM β-mercaptoethanol, 10 mM EDTA, 0.1% sodium lauryl sarcosine, 0.1% Triton X-100), and clarified by centrifugation for 10 min at 18,000 × *g*. After determining protein concentrations using the Bradford reagent (Thermo) with bovine serum albumin standards, 100 μl of 0.2 μg/μl protein suspensions were added to 100 μl of 2 mM 4-methylumbelliferyl-β-glucuronide in 96-well plates. After incubation at 37°C for 60 min, 25 μl of each reaction was added to 250 μl of cold stop buffer (0.2 M NaCO_3_ pH 9), and fluorescence measured using 365 nm excitation and 455 nm emission wavelengths. The significance of differences between classes and relative variance between individuals within classes were calculated by Student's T-test and F-test, respectively.

## Results

### Annotation of ribosomal protein genes

Using searches for genes classified under the GO term "ribosome" and BLASTP queries with ribosomal proteins from other species, 85 genes encoding 79 cytosolic ribosomal proteins were identified from the T30-4 reference genome of *P*. *infestans*. These represent the full complement of ribosomal proteins that are well-conserved among eukaryotes, including 32 from the 40S subunit and 47 from the 60S subunit. Two were encoded by sequences not previously identified as genes in the *P*. *infestans* reference genome. A similar approach identified orthologs from *Phytophthora capsici* and *Phytophthora parasitica*. The number of genes encoding each protein from the three *Phytophthora* species, diatoms, plants, fungi, yeast, and mammals are shown in S2 Table.

While many genes in *Phytophthora* belong to small families [37], this was not generally true for those encoding ribosomal proteins. In *P*. *infestans*, all proteins were encoded by single genes except for six components of the large subunit (L5, L6, L14, L40, LP1 and LP2). The use of single genes for most ribosomal proteins is typical for eukaryotes, except for plants and some fungi (S2 Table; [30]). In *P*. *infestans*, the duplicated genes encoding L6, LP1, and LP2 were also present in two copies in *P*. *capsici* and *P*. *parasitica*.

Most genes were distributed throughout the *P*. *infestans* genome. However, those encoding L22 and LP2 (i.e. *PiRLP22* and *PiRPLP2*) were adjacent to each other and transcribed from a common promoter region. The same relationship exists between L5 and L15. Also near each other were two *PiRPL40* genes (separated by three genes or 4.2 kb), *PiRPL27* and *PiRPL35A* (separated by four genes or 4.6 kb), and S26 and L39 (separated by five genes or 11.4 kb).

Compared to other *P*. *infestans* genes, a disproportionate number of those encoding ribosomal proteins (62%) contained introns towards the 5' ends of the primary transcript. The introns most commonly resided within 10 nt of start codon, which is a very distinct distribution (*P* = 10^−12^) from that observed for introns in other *P*. *infestans* genes (S1 Fig). This phenomenon has also been seen in other taxa, where some introns were shown to encode small nucleolar RNAs (snoRNA; [38]). The introns may also enhance expression of the genes. Using the SnoGPS program [33] with *P*. *infestans* rRNA as a target, we predicted pseudouridylation-guide snoRNAs in about half of the cases where introns occurred in the 5' portion of the primary transcript.

### Promoter motifs shared by ribosomal protein genes

**Fig 1:**
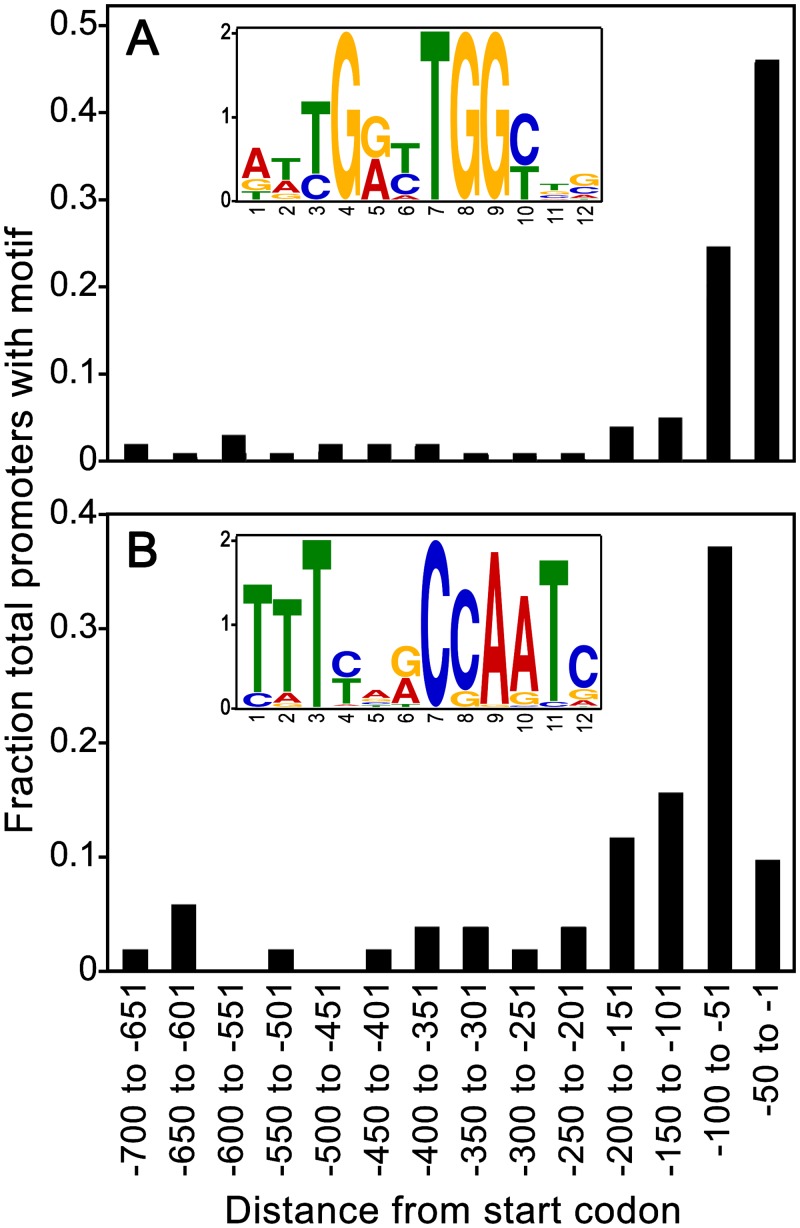
Motifs in ribosomal protein gene promoters. (**A)** distribution of PhRiboBox upstream of ribosomal protein genes. A logoplot of the motif is indicated in the box. (**B)** same as panel A except showing CCAAT element.

To learn if the *P*. *infestans* genes encoding ribosomal proteins bear a common transcription factor binding site, their promoters were searched for over-represented motifs using MEME. A new motif of 12-nt was detected in 68 of the 85 promoters and named the PhRiboBox (Fig 1A; S2 Fig). It was overrepresented significantly in promoters of genes encoding ribosomal proteins compared to total promoters (*p* = 10^−35^). The PhRiboBox was found at a median distance of 64-nt upstream of the start codon, and in forward and reverse orientations at similar rates. Its location was often evolutionarily conserved, as shown in S3 Fig for the *P*. *infestans* gene encoding ribosomal protein S9 *(PiRPS9)* and orthologs from three related species. Although the PhRibobox is just upstream of the transcription start site of *PiRPS9*, this is not the case for all ribosomal protein genes, as the motif was found up- and downstream of the major transcription start site at similar frequencies. The PhRiboBox lacks similarity to motifs known to regulate many ribosomal protein genes in fungi and metazoans [39, 40].

A second motif resembled the eukaryotic CCAAT box (Fig 1B). This was present in 83 of the 85 ribosomal promoters, which was much more frequent than in total promoters (*p* = 10^−66^). The CCAAT box, which is present in 10.9% of total promoters in *P*. *infestans*, has been shown to be associated with housekeeping genes and is usually close to the core promoter [41]. Under-represented in the ribosomal protein promoters were the three core motifs (INR, FPR, and DPE) previously identified in *P*. *infestans* [41].

Gene Ontology (GO) analysis indicated that the PhRiboBox was not just over-represented in genes encoding ribosomal proteins, but also in many other genes involved in ribosome biogenesis, translation, transcription, and DNA replication (Table 1). For example, 124 genes containing the PhRiboBox were classified under the term "nucleic acid binding". The *p*-value for over-representation of this category was 2 × 10^−10^. Proteins encoded by genes in this group included DNA-directed RNA polymerases, proteins involved in rRNA and tRNA maturation, translation initiation and elongation factors, DNA polymerases, histones, and regulators of DNA replication (Table 2). This analysis focused on genes containing the PhRiboBox within 200-nt of their start codon, to help reduce the number of false positives.

**Table 1:** Over-represented Gene Ontology terms in genes containing PhRiboBox.

Term	Definition	*P*-value	Number of genes
GO:0003735	Structural component of ribosome	6e-29	40
GO:0006412	Translation	3e-24	64
GO:0003723	RNA binding	8e-20	43
GO:0010467	Gene expression	2e-19	110
GO:0004249	Cellular biosynthesis	1e-17	100
GO:0003676	Nucleic acid binding	2e-10	124
GO:0042254	Ribosome biogenesis	7e-9	16
GO:0019538	Protein metabolic process	1e-5	134
GO:0008135	Translation factor	1e-4	18
GO:0006396	RNA processing	4e-4	11

Shown are Molecular Function and Biological Process terms based on a threshold of *p*<10^−3^, after removing most redundant terms. The search was based on genes containing the motif within 200-nt of start codon.

**Table 2:** Selected genes with PhRiboBox with GO:0003676 (nucleic acid binding).

Role	Definition
RNA synthesis	RNA polymerase subunits (RPA190, RPA135, RPC40, RPB5, RPB3, RPC34)
	Poly(A) polymerase
RNA modification	pre-mRNA-splicing factor PRP16
	pre-rRNA-splicing factor MRD1
	pre-rRNA-processing protein ESF2
	tRNA (adenine-N1)-methyltransferase
	tRNA (cytosine-5-)-methyltransferase
	tRNA pseudouridine synthase
	rRNA 2'-O-methyltransferase
	rRNA cytosine-5-methyltransferase
	rRNA processing exonuclease RRP44
	RNA helicase (12 genes)
Translation	Initiation factors (eIF1B, eIF2A, eIF2B, eIF3B, eIF3C, eIF3H, eIF4A, eIF4E)
	Elongation factors (eEF1A, eEF1G, G, P, Tu)
	Polypeptide release factor
	Polyadenylate-binding protein
DNA replication	DNA polymerase subunits (alpha/epsilon B, epsilon catalytic, kappa, lambda)
	Histones (H1, H2A, H2B, H3, H4)
	DNA replication licensing factor (MCM5, MCM7)
	Proliferating cell nuclear antigen
	DNA repair protein (REV1, RAD51, RAD23)

Genes are listed if they contain the PhRiboBox in the 200-nt upstream of the start codon. Nomenclature of RNA polymerases and DNA repair proteins follow the convention of the *Saccharomyces cerevisiae* genome database. A complete list of the genes is shown in S3 Table.

### Candidate promoters for driving transgenes

**Fig 2:**
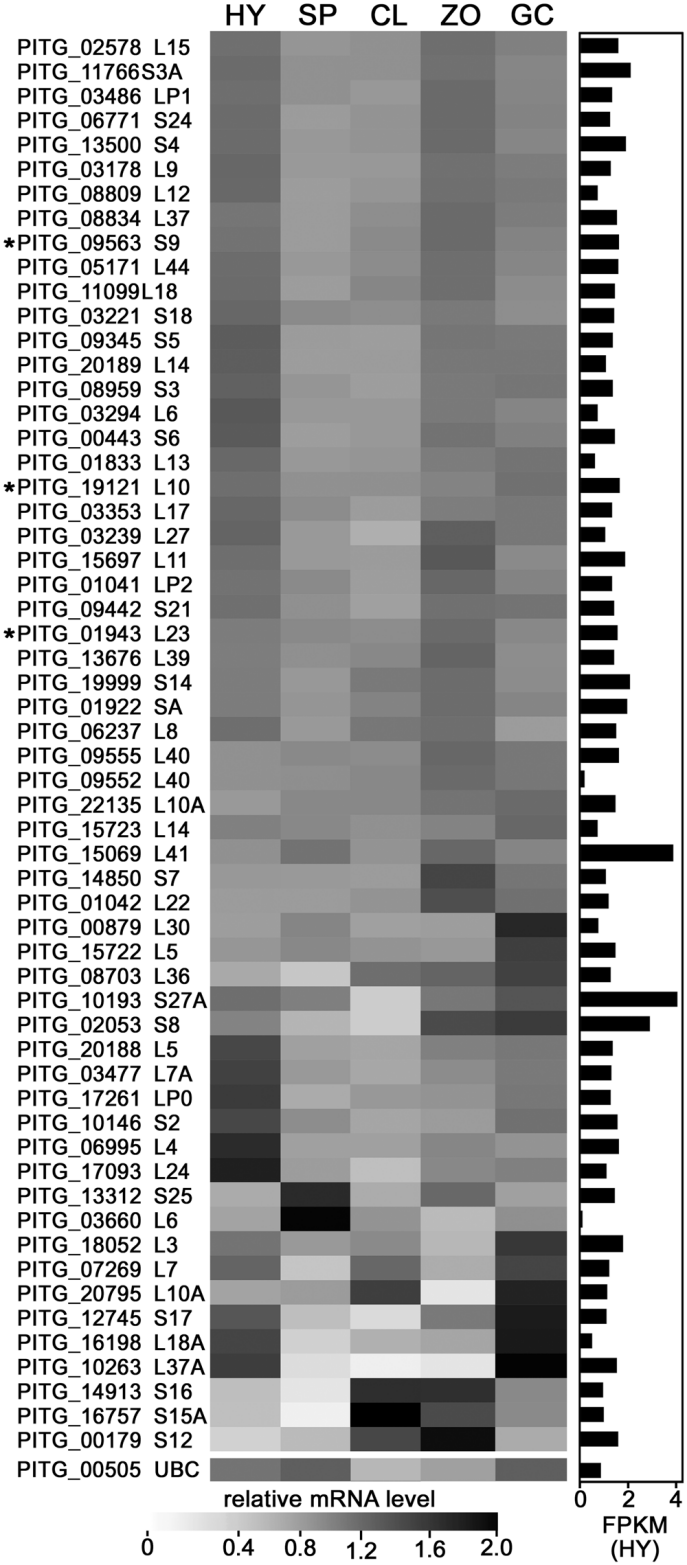
Transcription of *P*. *infestans* genes encoding ribosomal proteins. The heat map shows the per-gene normalized expression, based on microarrays, of the genes in nonsporulating hyphae (HY), sporangia (SP), sporangia chilled to induce their cleavage into zoospores (CL), motile zoospores (ZO), and germinated cysts forming appressoria (GC). The genes are ordered based on their expression stability, with the most invariant at the top. The bar graph indicates relative expression (FPKM) in hyphae based on RNA-seq. The three genes marked by asterisks are the donors of promoters for the expression studies described later in Results. Genes PITG_09552 and PITG_09555 are nearly identical in sequence and not distinguished well by the microarray. For comparison, shown at the bottom of the chart is PITG_00505, which encodes an ubiquitin-conjugating enzyme; a prior study of putative housekeeping genes ranked its *Phytophthora parasitica* ortholog at the top of genes having the most consistent mRNA levels during growth and development [42].

Good promoters for expressing transgenes should be transcribed at similar levels and fairly high levels in all tissue types. To identify ribosomal protein genes with constitutive expression, we examined existing microarray data for hyphae, sporangia, germinated sporangia, zoospores, and germinating zoospore cysts [34]. About one-half of the genes showed only minor variation from the mean between the different life-stages (Fig 2). Only about two-thirds of the genes were represented on the array and gave reliable signals; the lack of data from some genes is not a concern since many are likely expressed at low levels and not optimal for expressing transgenes.

To accurately measure mRNA levels of the genes, we generated RNA-seq data from hyphae grown in rye-sucrose broth. Most ribosomal protein genes were expressed at high levels. Their average FPKM was 1689, which was 37-fold higher than average and in the top 1% of all genes (Fig 2). This is similar to the expression level of ribosomal genes in other taxa [25]. Very few RNA reads mapped to PITG_03660 and PITG_09552, which are predicted to encode L6 and L40, respectively. These may be unexpressed pseudogenes, since other *PiRPL6* and *PiRPL40* genes are present which had high FPKM levels. PITG_03660 likely represents a fairly old pseudogene since two L6-like genes are also found in *P*. *capsici* and *P*. *parasitica*. The genes encoding L14 and LP2 are also duplicated in all three species, but each appears to be functional with high FPKM values. Pseudogenes for ribosomal proteins are fairly common in other species [43].

There was no correlation between the mRNA level of each gene and whether it contained an intron (*p* = 0.36), PhRiboBox (*p* = 0.45), or CCAAT element (*p* = 0.40). There was also no correlation between the relative stability of gene expression in the five different life-stages and those features (*p* = 0.34, 0.45, and 0.47, respectively).

Based on the above, we chose genes encoding ribosomal proteins L23, L10, and S9 as sources of promoters for further study. These correspond to *P*. *infestans* genes PITG_01943, PITG_19121 and PITG_09563, and *P*. *capsici* genes PHYCA_93109, PHYCA_91078, and PHYCA_89970. All have constitutive expression (standard deviation between life-stages <15%), high mRNA levels, and well-supported gene models. The *RPS9* promoters have both the PhRiboBox and CCAAT motif, *RPL10* has the CCAAT motif only, and *RPL23* has only the PhRiboBox within 200-nt of the start codon.

### Strength of transgene expression using ribosomal promoters

**Fig 3:**
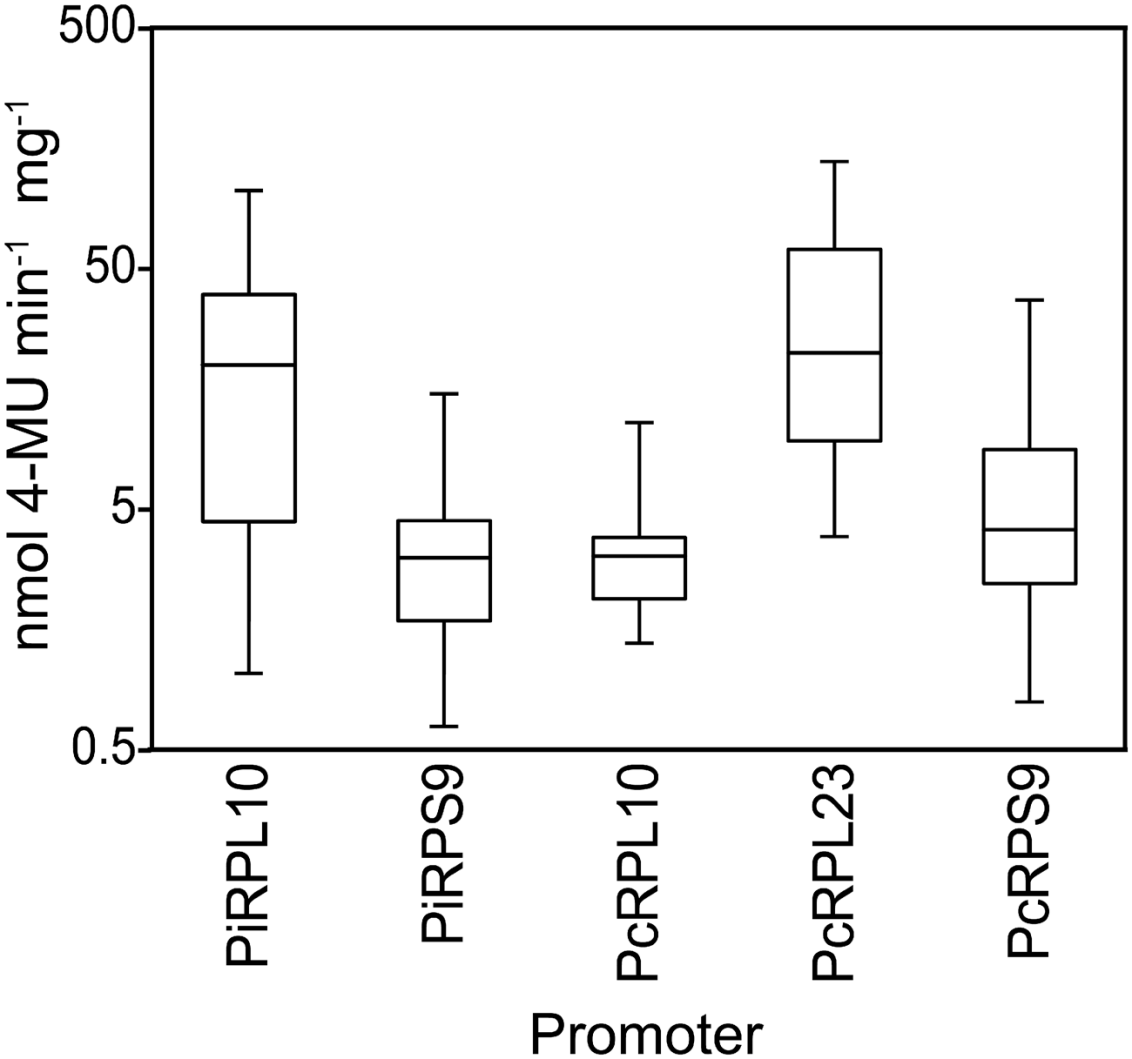
GUS expression driven by five ribosomal protein promoters in stable transformants. The box plots for each gene reflect the distribution of activities from a minimum of ten independent transformants for each construct, which employed 500-nt promoter fragments from *P*. *infestans* (Pi) or *P*. *capsici* (Pc) genes. Expression driven by the *PiRPL23* promoter (PiL23) is not shown due to its instability.

The six promoters were tested in stable transformants of *P*. *infestans* using the GUS reporter (Fig 3). This involved placing 500-nt of sequences upstream of their start codons in front of a promoter-less GUS reporter gene, in a plasmid backbone that confers resistance to G418. This size was chosen since the median intergenic distance in *P*. *infestans* is 430-nt [44]. The overall fraction of G418-resistant transformants that initially stained positive for GUS was 42%, and we did not observe a significant difference between plasmids or promoters except for the *PiRPL23* construct. Less than 10% of primary transformants with the *PiRPL23* promoter exhibited GUS activity, and most stopped growing after one or two transfers. The *PiRPL23* promoter sequences may have been lethal, perhaps by triggering silencing of an essential native gene.

Quantitative assays using transformants obtained with the five other plasmids revealed that GUS activity varied substantially between strains using the same promoter, probably due to position or copy number effects. Position effects in *P*. *infestans*, which has a large repeat-rich genome, have previously been described [45]. The strongest expression resulted from the *PiRPL10* and *PcRPL23* promoters, but there was no correlation between orthologs. For example, *PiRPL10* was the strongest *P*. *infestans* promoter while *PcRPL10* was the weakest *P*. *capsici* promoter. These results could be biased, since the quantitative assays were applied just to strains that exhibited positive histochemical staining.

### Transgene stability using ribosomal promoters

**Fig 4:**
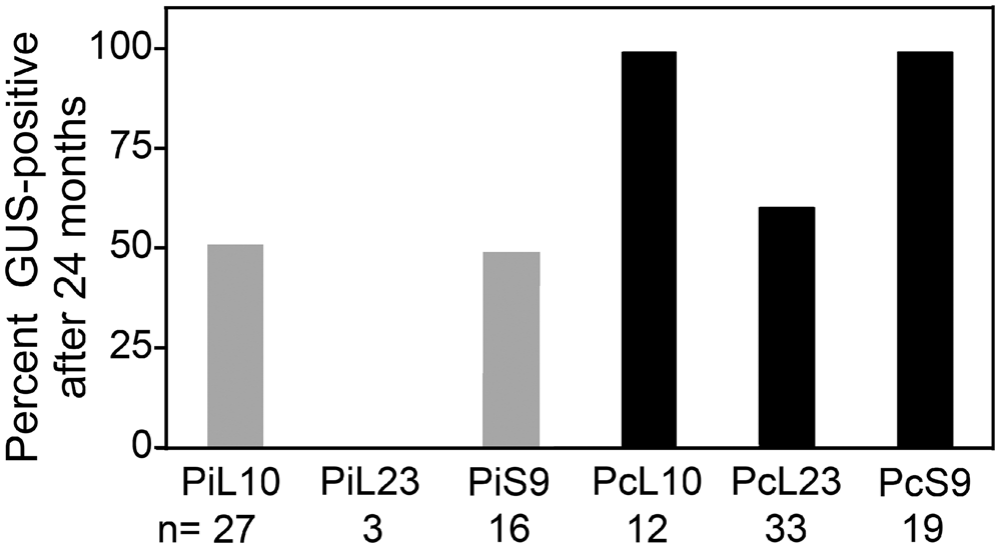
Stability of GUS expression in transformants. The bars represent the percentage of clones still expressing GUS after 24 months. Indicated below each bar is the promoter name and the number of transformants analyzed.

GUS expression in *P*. *infestans* was more stable using *P*. *capsici* promoters than their *P*. *infestans* counterparts (Fig 4). This involved subjecting strains that had initially exhibited activity in colonies scored on the primary transformant selection plates to histochemical staining on a monthly basis for two years. While expression persisted in all 12 *PcL10* transformants during the two years, about half of the 27 transformants using *PiRPL10* lost visible activity. Similarly, while GUS activity remained in all 19 *P*. *infestans* transformants using *PcRPS9*, nearly half of the 16 employing *PiRPS9* lost expression. This indicates that stability was much higher in the *P*. *infestans* transformants using *P*. *capsici* versus *P*. *infestans* regulatory sequences (*p* = 10^-7^). *P*. *capsici* promoters were not altogether immune to silencing, since activity was lost from 9 of 33 transformants using *PcRPL23*.

With the *P*. *infestans* promoters, the cessation of GUS expression progressed throughout the two year experiment. For example, five transformants with the *PiRPL10* promoter lost GUS expression after 1–3 months, four after 4–7 months of culture, and four after a total of 12 months. Similarly, expression from the *PiRPS9* promoter ceased in five transformants within the first three months, and three more afterwards. PCR assays of selected transformants indicated that strains that lost GUS activity had maintained the transgene. This implicated epigenetic events as the main cause of expression instability.

The stability of the *ham34* promoter was not examined in side-by-side experiments with the ribosomal gene promoters. However, we conducted similar studies in 1995 and 2012. The stability of GUS expression in those experiments were 68% and 75%, respectively, over a two year period.

### Use of promoters does not affect native gene expression

**Fig 5:**
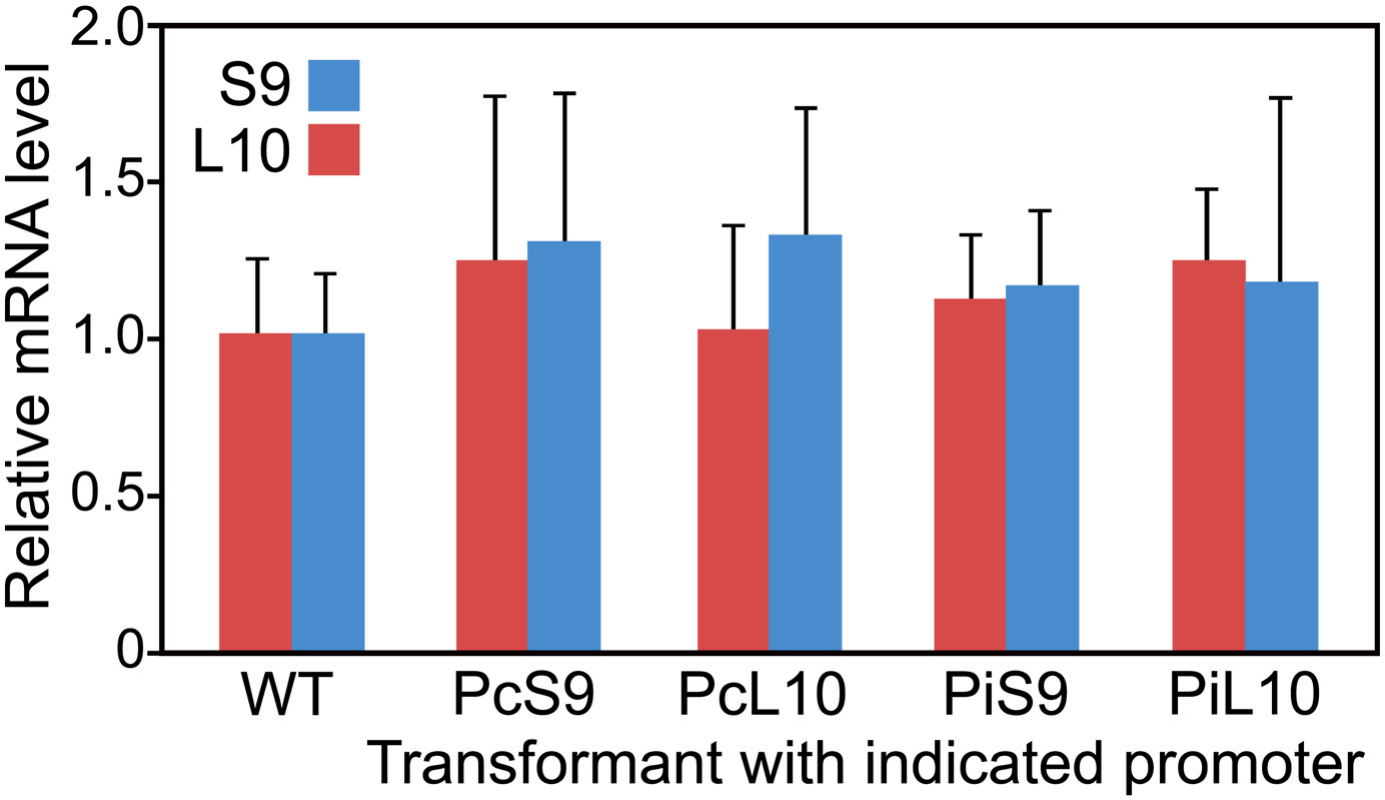
Expression of native *PiRPL10* and *PiRPS9* genes in transformants bearing transgenes with *RPL10* and *RPS9* promoters. mRNA levels were determined by RT-qPCR of cultures grown in rye-sucrose broth, and are expressed relative to the level in untransformed strain 1306 (WT). Error bars reflect variation in three biological replicates.

Employing a promoter to express transgenes has occasionally been reported to affect related promoters, possibly due to titration of transcription factors [46]. We tested this possibility by using RT-qPCR to measure the expression of the native *PiRPL10* and *PiRPS9* genes in transformants expressing GUS behind promoters from *PiRPL10*, *PcRPL10*, *PiRPS9*, and *PcRPS9* (Fig 5). The results indicated that using these promoters did not significantly alter the expression level of the corresponding genes. For example, the expression of neither *PiRPL10* or *PiRPS9* was significantly different in the wild type progenitor strain compared to transformants expressing GUS fused to the *PiRPS9* or *PcRPS9* promoters (*P*>0.3 and *P*>0.5, respectively).

### New vectors for overexpression

**Fig 6:**
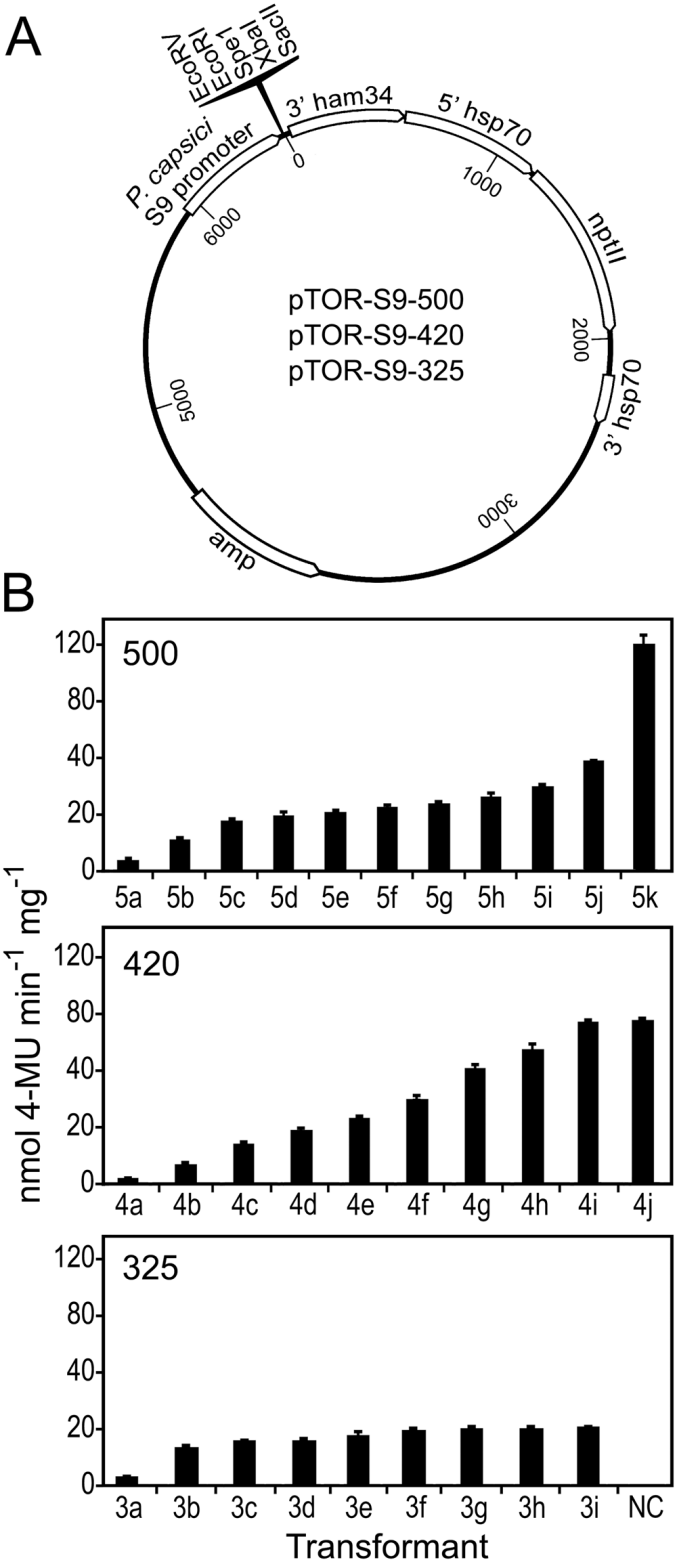
Effect of *PcRPS9* promoter size on GUS expression. (**A)** Expression vectors based on the 500, 420, and 325-nt versions of the *PcRPS9* promoter. (**B)** Expression driven by different versions of the *PcRPS9* promoter in *P*. *infestans*. Each bar represents values from independent transformants, based on the average of two biological replicates. NC is an empty-vector control.

We chose to develop an expression vector using *PcRPS9* regulatory sequences since it resulted in higher average GUS levels than the *PcRPL10* promoter, although both resulted in durable expression. First, we exchanged the *PcRPS9* promoter for the *B*. *lactucae ham34* promoter in pTOR, a vector that is used widely by the oomycete community (Fig 6A). We also inserted a GUS gene into the plasmid to allow us to test 500, 420, and 325-nt versions of the *PcRPS9* promoter. The smaller *PcRPS9* promoters were tested in an attempt to reduce vector size and minimize unneeded sequences that might affect stability. *PcRPS9* transcription is predicted to start about 30-nt upstream of the 3' end of the promoter.

Quantitative assays (Fig 6B) of transformants obtained using the three promoter-GUS fusion plasmids indicated that the mean levels of GUS obtained with the 500 and 420-nt *PcRPS9* promoters were not significantly dissimilar (*p* = 0.46). However, they enabled an average of 74% higher activity than the 325-nt version, which is a significant difference (*p* = 0.04). While the 325-nt promoter yielded less average activity, it was curious that it resulted in less variation between transformants compared to the longer fragments (*p* = 0.05). The same conclusions were drawn when the transformants were assayed using the semi-quantitative histochemical assay for GUS.

**Fig 7:**
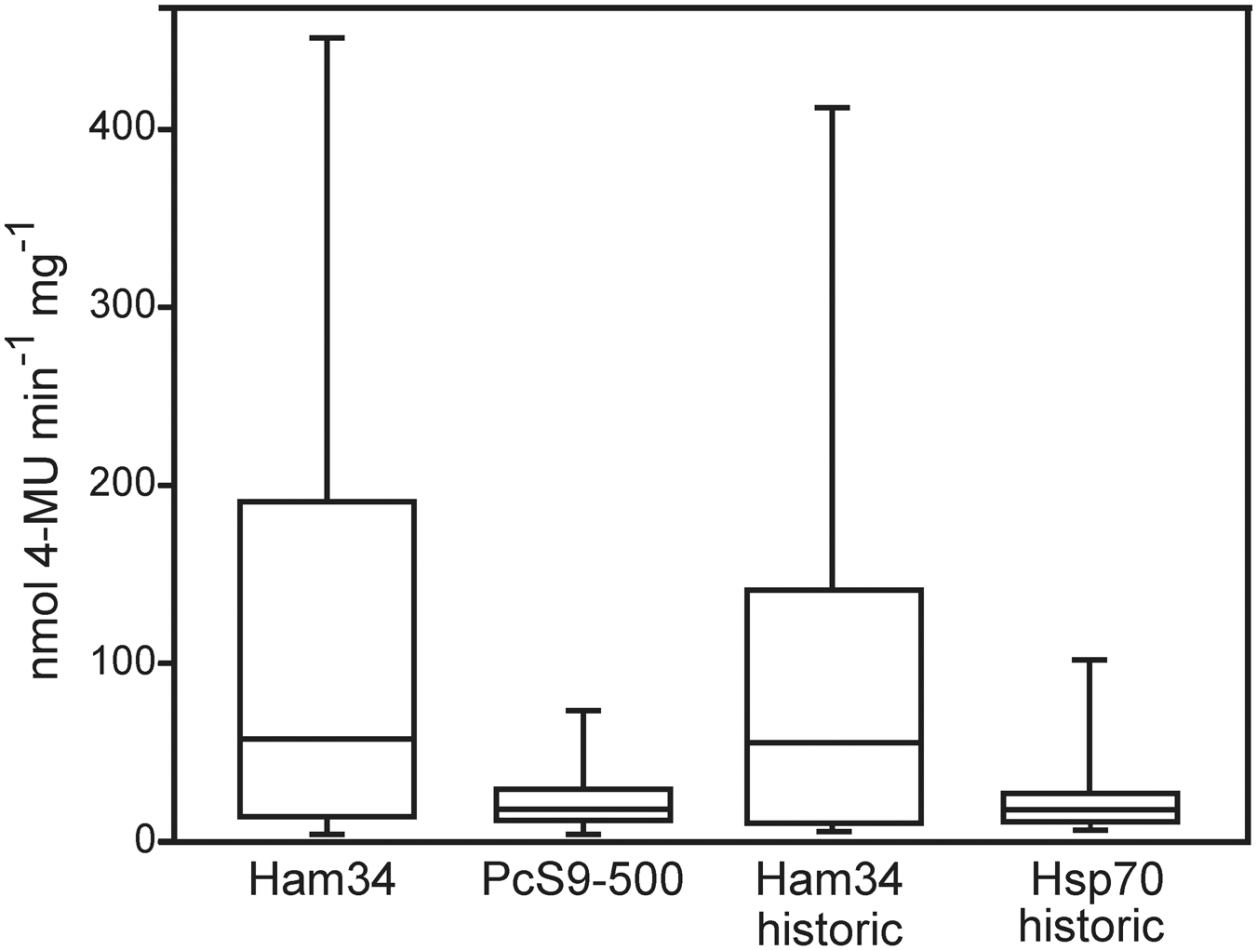
Comparison of *PcRPS9* and *ham34* promoter strength. Transformants of *P*. *infestans* were obtained in parallel experiments using plasmids containing the two promoters fused to the GUS gene. Specific activities of the transformants were then determined. Also shown are historic data for GUS driven by *ham34* and *hsp70*, which was taken from reference 45. The middle line in the box plot represents the median expression level.

To compare the level of GUS expression achieved with the 500 nt *PcRPS9* promoter and the *ham34* promoter of *B*. *lactucae*, we generated twenty additional transformants of *P*. *infestans* using those two promoters. Quantitative assays indicated that the median expression with *PcRPS9* was 26% of that obtained with *ham34* (Fig 7). The figure also shows GUS levels measured in a prior study of *ham34* [45], which are nearly identical to the new data generated here. Fig 7 also shows that the levels obtained with *PcRPS9* are nearly identical to those reported in that prior study for the *hsp70* promoter of *B*. *lactucae*, which has proved to be strong in *P*. *infestans*.

### Promoter activity *in planta*

**Fig 8:**
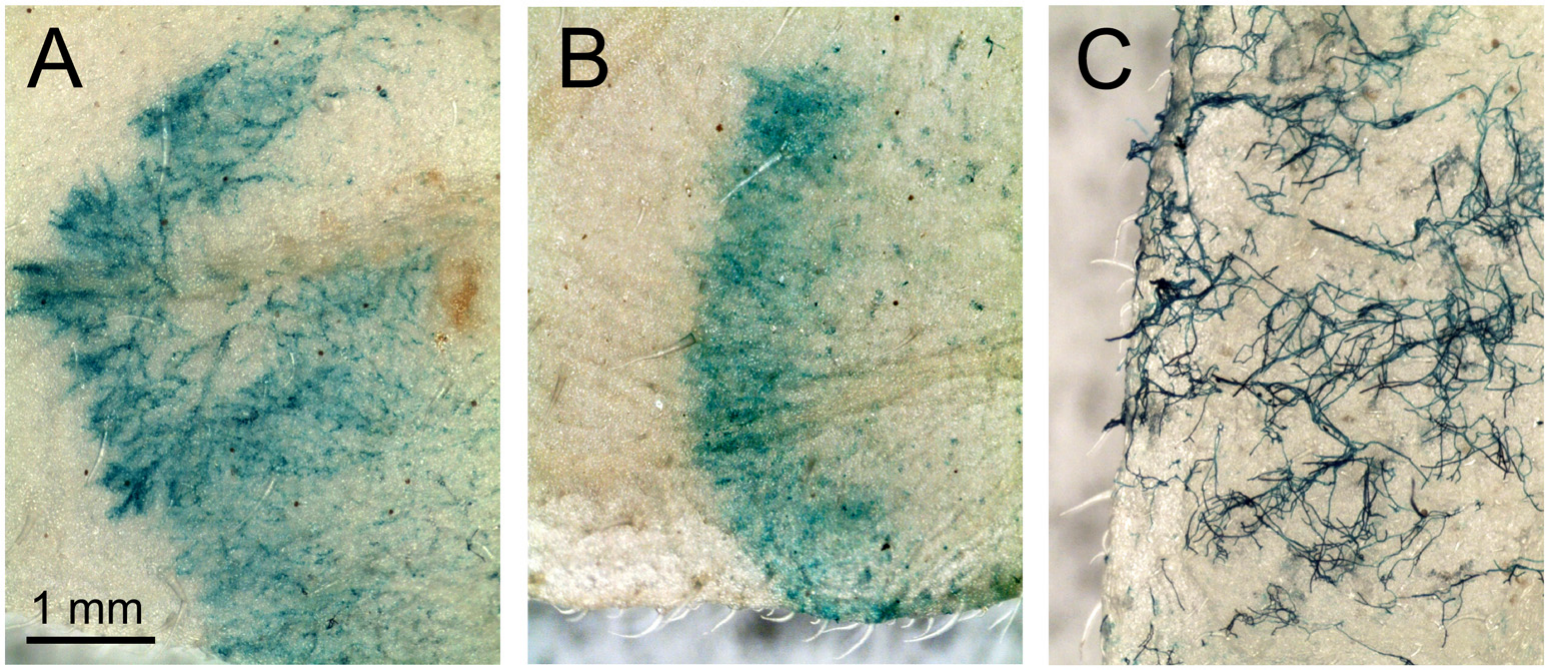
*In planta* expression of *PcRPS9* promoter. Detached tomato leaflets were inoculated with a *P*. *infestans* transformant expressing a fusion between GUS and the promoter from *PcRPS9*, and stained histochemically after 4 days. **(A**, **B)** edges of lesions in which *P*. *infestans* was growing within the leaflet. The direction of growth is from right to left. Little staining is observed in the older part of the lesion, since the hyphae there have become vacuolated. (**C)** region of leaflet where sporulation was starting, showing staining of hyphae emerging on the plant surface.

To confirm that the *PcRPS9* promoter was expressed during plant infection, transformants were inoculated on tomato leaflets and stained for GUS. Expression was observed both in hyphae within the plant and on surface hyphae at 4 days post-infection (Fig 8). Similar results were obtained with transformants in which GUS was driven by the *PcRPL10* promoter (not shown).

Near the conclusion of the study, we obtained RNA-seq data for *P*. *infestans* in tomato leaflets. This enabled the activities of the native *PiRPS9*, *PcRPL10*, and *PcRPL23* promoters to be assessed *in planta*. As shown in S4 Fig, all three genes are expressed strongly during plant infection. Their RNA levels were slightly higher and lower at early (3 day) and late (6 day) infection stages, respectively, than in rye media. This minor variation should not detract from the utility of the promoters for expressing transgenes.

## Discussion

A prerequisite for modern genetics is a versatile toolkit for manipulating an organism's genes. Our study has expanded such tools for oomycetes by identifying new promoters, thus providing researchers with more options for designing plasmids and stacking transgenes. Numerous promoters for expressing transgenes are available from plants, animals, and fungi, but regulatory sequences from non-oomycetes function poorly in *Phytophthora* [47]. We therefore focused on identifying promoters from *P*. *infestans* and *P*. *capsici*. Only some of their ribosomal promoter genes were appropriate donors of promoters, since as in other taxa [48–50] many are developmentally regulated. Our functional tests in *P*. *infestans* indicated that the regulatory sequences from the *PcRPS9* and *PcRPL10* genes conferred high-level expression of the GUS reporter, which remained stable over at least two years. The *P*. *infestans* orthologs also exhibited strong activity in *P*. *infestans*, but with lower stability. We expect that the *PcRPS9* and *PcRPL10* promoters will also be useful for other oomycetes, although groups studying *P*. *capsici* may wish to test the *P*. *infestans* orthologs.

Several models may explain the higher stability of expression from the *PcRPS9* and *PcRPL10* sequences in *P*. *infestans* compared to the other promoters. For example, the *P*. *infestans* orthologs may be more likely to knock-down the native ribosomal protein genes through RNA interference triggered by their shared 5' untranslated regions (UTRs). Instability may also result from silencing caused by antisense UTR RNAs generated from cryptic transcription start sites within the *P*. *infestans* promoters [51]. In either scenario, there would be pressure to silence the transgene, since altering ribosomal protein expression should be deleterious [43]. The situation observed with the transgene resembles that described for natural isolates of *P*. *sojae*, where plant resistance genes are hypothesized to select for epigenetic silencing of avirulence genes in the pathogen [52]. It is unlikely that the instability seen with the *P*. *infestans* promoters resulted from their recombination with the native ribosomal protein genes, since homologous integration during *P*. *infestans* transformation is rare [24].

It is notable that the degree of persistence of expression from *PcRPS9* and *PcRPL10* promoters seemed higher than that reported for the *ham34* or *hsp70* promoters from *B*. *lactucae* [45]. Those experiments were performed using the same isolate of *P*. *infestans* and transformation protocol that was employed in our present study. It is possible that the two *P*. *capsici* promoters fortuitously contain sequences that inhibit silencing. In mammals, some satellite DNAs and CpG-depleted regions help maintain transcriptionally permissive chromatin in nearby transgenes [53, 54]. Portions of transposable elements have also proved to suppress the silencing of transgenes in plants [55]. We have not detected such sequences within the *PcRPS9* and *PcRPL10* promoters, but others that affect expression stability may be present.

It is also possible that the high stability of *PcRPS9* and *PcRPL10*-driven transcription relates to their lack of an INR-FPR core promoter element [41]. The two *B*. *lactucae* promoters both contain an INR-FPR motif, which tends to be found in oomycete genes that are regulated developmentally [41]. Chromatin around those promoters may be more prone to remodeling and the imposition of transcriptional quiescence than the sequences that regulate most ribosomal protein genes. In metazoans, a TCT element is found upstream of many ribosomal protein genes and is associated with very low nucleosome occupancy [40]. It is possible that the PhRiboBox produces a similar result.

## Supporting Information

S1 Fig Location of introns in ribosomal protein genes and total *P*. *infestans* genes. (PDF)

S2 Fig Probability matrix for PhRiboBox. (PDF)

S3 Fig Conservation of PhRiboBox in orthologs of *P*. *infestans* gene encoding ribosomal protein S9. (PDF)

S4 Fig Relative expression of *PiRPL10*, *PiRPL23*, *and PiRPS9* in rye media and during tomato leaflet infection. (PDF)

S1 Table Primers used for cloning promoter regions. (PDF)

S2 Table Number of genes encoding ribosomal proteins in *Phytophthora* spp. and other eukaryotes. (PDF)

S3 Table *P*. *infestans* genes containing the PhRiboBox in the 200-nt upstream of their start codons with GO annotation GO:0003676 for Nucleic Acid Binding. (PDF)

## References

[1] Kamoun S, Furzer O, Jones JD, Judelson HS, Ali GS, Dalio RJ, et al The Top 10 oomycete pathogens in molecular plant pathology. Molec Plant Pathol. 2015;16: 413–434. 25178392 10.1111/mpp.12190 PMC6638381

[2] Judelson HS, Whittaker SL. Inactivation of transgenes in *Phytophthora infestans* is not associated with their deletion, methylation, or mutation. Curr Genet. 1995;28: 571–579. 8593689 10.1007/BF00518171

[3] Xiang QJ, Judelson HS. Myb transcription factors and light regulate sporulation in the oomycete *Phytophthora infestans* . PloS One. 2014;9: e92086 10.1371/journal.pone.0092086 24704821 PMC3976263

[4] Panabieres F, Birch PR, Unkles SE, Ponchet M, Lacourt I, Venard P, et al Heterologous expression of a basic elicitin from *Phytophthora cryptogea* in *Phytophthora infestans* increases its ability to cause leaf necrosis in tobacco. Microbiology. 1998;144: 3343–3349. 9884226 10.1099/00221287-144-12-3343

[5] Matzke AJM, Matzke MA. Position effects and epigenetic silencing of plant transgenes. Curr Opin Plant Biol. 1998;1: 142–148. 10066569 10.1016/s1369-5266(98)80016-2

[6] Ellis J. Silencing and variegation of gammaretrovirus and lentivirus vectors. Hum Gene Ther. 2005;16:1241–1246. 16259557 10.1089/hum.2005.16.1241

[7] Scrable H, Stambrook PJ. A genetic program for deletion of foreign DNA from the mammalian genome. Mutat Res-Fund Mol M. 1999;429: 225–237. 10.1016/s0027-5107(99)00114-1 10526207

[8] Garrick D, Fiering S, Martin DIK, Whitelaw E. Repeat-induced gene silencing in mammals. Nature Genet. 1998;18: 56–59. 9425901 10.1038/ng0198-56

[9] Daxinger L, Hunter B, Sheik M, Jauvion V, Gasciolli V, Vaucheret H, et al Unexpected silencing effects from T-DNA tags in Arabidopsis. Trends Plant Sci. 2008;13: 4–6. 10.1016/j.tplants.2007.10.007 18178509

[10] Agapito-Tenfen SZ, Vilperte V, Benevenuto RF, Rover CM, Traavik TI, Nodari RO. Effect of stacking insecticidal *cry* and herbicide tolerance *epsps* transgenes on transgenic maize proteome. BMC Plant Biol. 2014;14: 346 10.1186/s12870-014-0346-8 25490888 PMC4273480

[11] Bhullar S, Chakravarthy S, Advani S, Datta S, Pental D, Burma PK. Strategies for development of functionally equivalent promoters with minimum sequence homology for transgene expression in plants: cis-elements in a novel DNA context versus domain swapping. Plant Physiol. 2003;132:988–998 12805627 10.1104/pp.103.020602 PMC167037

[12] Judelson HS, Michelmore RW. Highly abundant and stage-specific messenger RNA in the obligate pathogen *Bremia lactucae* . Molec Plant-Microbe Interact. 1990;3: 225–232. 2131094 10.1094/mpmi-3-225

[13] Feng BZ, Zhu XP, Fu L, Lu RF, Storey D, Tooley P, et al Characterization of necrosis-inducing NLP proteins in *Phytophthora capsici* . BMC Plant Biol. 2014;14: 126 10.1186/1471-2229-14-126 24886309 PMC4023171

[14] van West P, Reid B, Campbell TA, Sandrock RW, Fry WE, Kamoun S, et al Green fluorescent protein (GFP) as a reporter gene for the plant pathogenic oomycete *Phytophthora palmivora* . FEMS Microbiol Lett. 1999;178: 71–80. 10483725 10.1111/j.1574-6968.1999.tb13761.x

[15] Randall E, Young V, Sierotzki H, Scalliet G, Birch PRJ, Cooke DEL, et al Sequence diversity in the large subunit of RNA polymerase I contributes to Mefenoxam insensitivity in *Phytophthora infestans* . Molecular Plant Pathol. 2014;15: 664–676. 10.1111/mpp.12124 PMC6638662 24521429

[16] Thines M. Phylogeny and evolution of plant pathogenic oomycetes-a global overview. Eur J Plant Pathol. 2014;138: 431–47.

[17] Bottin A, Larche L, Villalba F, Gaulin E, Esquerre-Tugaye M-T, Rickauer M. Green fluorescent protein (GFP) as gene expression reporter and vital marker for studying development and microbe-plant interaction in the tobacco pathogen *Phytophthora parasitica* var. *nicotianae* . FEMS Microbiol Lett. 1999;176: 51–56. 10917747 10.1111/j.1574-6968.1999.tb13641.x

[18] Judelson HS, Michelmore RW. Structure and expression of a gene encoding heat-shock protein Hsp70 from the oomycete fungus *Bremia lactucae* . Gene. 1989;79: 207–218. 2792764 10.1016/0378-1119(89)90203-5

[19] Jupe J, Stam R, Howden AJM, Morris JA, Zhang RX, Hedley PE, et al Phytophthora capsici-tomato interaction features dramatic shifts in gene expression associated with a hemi-biotrophic lifestyle. Genome Biol. 2013;14: R63 10.1186/gb-2013-14-6-r63 23799990 PMC4054836

[20] Ah-Fong AM, Judelson HS. Vectors for fluorescent protein tagging in *Phytophthora*: tools for functional genomics and cell biology. Fungal Biol. 2011;115: 882–890. 10.1016/j.funbio.2011.07.001 21872185

[21] Gamboa-Melendez H, Judelson HS. Development of a bipartite ecdysone-responsive gene switch for the oomycete *Phytophthora infestans* and its use to manipulate transcription during axenic culture and plant infection. Molec Plant Pathol 16: 83–91. 24871323 10.1111/mpp.12161 PMC6638397

[22] Vetukuri RR, Avrova AO, Grenville-Briggs LJ, Van West P, Soderbom F, Savenkov EI, et al Evidence for involvement of Dicer-like, Argonaute and histone deacetylase proteins in gene silencing in *Phytophthora infestans* . Molec Plant Pathol. 2011;12: 772–85. 21726377 10.1111/j.1364-3703.2011.00710.x PMC6640358

[23] Judelson HS. Intermolecular ligation mediates efficient cotransformation in *Phytophthora infestans* . Molec Gen Genet. 1993;239: 241–250. 8510651 10.1007/BF00281624

[24] Van West P, Kamoun S, Van 't Klooster JW, Govers F. Internuclear gene silencing in *Phytophthora infestans* . Molec Cell. 1999;3: 339–348. 10198636 10.1016/s1097-2765(00)80461-x

[25] Horstick EJ, Jordan DC, Bergeron SA, Tabor KM, Serpe M, Feldman B, et al Increased functional protein expression using nucleotide sequence features enriched in highly expressed genes in zebrafish. Nucl Acids Res. 2015;43: e48 10.1093/nar/gkv035 25628360 PMC4402511

[26] Koia J, Moyle R, Hendry C, Lim L, Botella JR. Pineapple translation factor SUI1 and ribosomal protein L36 promoters drive constitutive transgene expression patterns in *Arabidopsis thaliana* . Plant Mol Biol. 2013;81: 327–36. 10.1007/s11103-012-0002-3 23263857

[27] Zhou J, Lin CZ, Zheng XZ, Lin XJ, Sang WJ, Wang SH, et al Functional analysis of an alpha-1,2-mannosidase from *Magnaporthe oryzae* . Current Genetics. 2009;55: 485–496. 10.1007/s00294-009-0261-y 19621226

[28] Dou D, Kale SD, Wang X, Chen Y, Wang Q, Wang X, et al Conserved C-terminal motifs required for avirulence and suppression of cell death by *Phytophthora sojae* effector Avr1b. Plant Cell. 2008;20:1118–33. 10.1105/tpc.107.057067 18390593 PMC2390733

[29] Stajich JE, Harris T, Brunk BP, Brestelli J, Fischer S, Harb OS, et al FungiDB: an integrated functional genomics database for fungi. Nucl Acids Res. 2012;40: D675–D81. 10.1093/nar/gkr918 22064857 PMC3245123

[30] Nakao A, Yoshihama M, Kenmochi N. RPG: the Ribosomal Protein Gene database. Nucl Acids Res. 2004;32: D168–D70. 14681386 10.1093/nar/gkh004 PMC308739

[31] Bailey TL, Bodén M, Buske FA, Frith M, Grant CE, Clementi L, et al MEME SUITE: tools for motif discovery and searching. Nucl Acids Res. 2009;37: W202–W8. 10.1093/nar/gkp335 19458158 PMC2703892

[32] Beissbarth T, Speed TP. GOstat: find statistically overrepresented Gene Ontologies within a group of genes. Bioinformatics. 2004;20: 1464–1465. 14962934 10.1093/bioinformatics/bth088

[33] Schattner P, Brooks AN, Lowe TM. The tRNAscan-SE, snoscan and snoGPS web servers for the detection of tRNAs and snoRNAs. Nucl Acids Res. 2005;33: W686–W9. 15980563 10.1093/nar/gki366 PMC1160127

[34] Judelson HS, Ah-Fong AM, Aux G, Avrova AO, Bruce C, Cakir C, et al Gene expression profiling during asexual development of the late blight pathogen *Phytophthora infestans* reveals a highly dynamic transcriptome. Mol Plant-Microbe Interact. 2008;21: 433–447. 10.1094/MPMI-21-4-0433 18321189

[35] Ah Fong A, Xiang Q, Judelson HS. Architecture of the sporulation-specific Cdc14 promoter from the oomycete *Phytophthora infestans* . Eukaryot Cell. 2007;6: 2222–2230. 17951514 10.1128/EC.00328-07 PMC2168256

[36] Ah-Fong AM, Bormann-Chung CA, Judelson HS. Optimization of transgene-mediated silencing in *Phytophthora infestans* and its association with small-interfering RNAs. Fungal Genet Biol. 2008;45: 1197–1205. 10.1016/j.fgb.2008.05.009 18599326

[37] Haas BJ, Kamoun S, Zody MC, Jiang RH, Handsaker RE, Cano LM, et al Genome sequence and analysis of the Irish potato famine pathogen *Phytophthora infestans* . Nature. 2009;461: 393–398. 10.1038/nature08358 19741609

[38] Amaldi F, Beccari E, Bozzoni I, Buongiornonardelli M, Pierandreiamaldi P, Tognoni A. Structure and expression of ribosomal protein genes in *Xenopus laevis* . Biochem Cell Biol. 1995;73: 969–977. 8722012 10.1139/o95-104

[39] Tanay A, Regev A, Shamir R. Conservation and evolvability in regulatory networks: The evolution of ribosomal regulation in yeast. Proc Nat Acad Sci USA. 2005;102:7203–7208. 15883364 10.1073/pnas.0502521102 PMC1091753

[40] Rach EA, Winter DR, Benjamin AM, Corcoran DL, Ni T, Zhu J, et al Transcription Initiation patterns indicate divergent strategies for gene regulation at the chromatin level. PLoS Genet. 2011;7: e1001274 10.1371/journal.pgen.1001274 21249180 PMC3020932

[41] Roy S, Poidevin L, Jiang T, Judelson HS. Novel core promoter elements in the oomycete pathogen *Phytophthora infestans* and their influence on expression detected by genome-wide analysis. BMC Genom. 2013;14: 106. 10.1186/1471-2164-14-106 PMC3599244 23414203

[42] Yan HZ, Liou RF. Selection of internal control genes for real-time quantitative RT-PCR assays in the oomycete plant pathogen *Phytophthora parasitica* . Fungal Genet Biol. 2006;43:430–438. 16531084 10.1016/j.fgb.2006.01.010

[43] Marygold SJ, Roote J, Reuter G, Lambertsson A, Ashburner M, Millburn GH, et al The ribosomal protein genes and Minute loci of *Drosophila melanogaster* . Genome Biol. 2007;8: R216 17927810 10.1186/gb-2007-8-10-r216 PMC2246290

[44] Roy S, Kagda M, Judelson HS. Genome-wide prediction and functional validation of promoter motifs regulating gene expression in spore and infection stages of *Phytophthora infestans* . Plos Pathog. 2013;9(3):e1003182 10.1371/journal.ppat.1003182 23516354 PMC3597505

[45] Judelson HS, Dudler R, Pieterse CMJ, Unkles SE, Michelmore RW. Expression and antisense inhibition of transgenes in *Phytophthora infestans* is modulated by choice of promoter and position effects. Gene. 1993;133:63–69. 8224895 10.1016/0378-1119(93)90225-r

[46] Natesan S, Rivera VM, Molinari E, Gilman M. Transcriptional squelching re-examined. Nature. 1997;390:349–350. 10.1038/37019 9389473

[47] Judelson HS, Tyler BM, Michelmore RW. Regulatory sequences for expressing genes in oomycete fungi. Molec Gen Genet. 1992;234:138–146. 1495476 10.1007/BF00272355

[48] Hughes TR, Marton MJ, Jones AR, Roberts CJ, Stoughton R, Armour CD, et al Functional discovery via a compendium of expression profiles. Cell. 2000;102:109–126. 10929718 10.1016/s0092-8674(00)00015-5

[49] Le Roch KG, Zhou YY, Blair PL, Grainger M, Moch JK, Haynes JD, et al Discovery of gene function by expression profiling of the malaria parasite life cycle. Science. 2003;301: 1503–1508. 12893887 10.1126/science.1087025

[50] Barakat A, Szick-Miranda K, Chang IF, Guyot R, Blanc G, Cooke R, et al The organization of cytoplasmic ribosomal protein genes in the Arabidopsis genome. Plant Physiol. 2001;127: 398–415. 11598216 PMC125077

[51] Neil H, Malabat C, d'Aubenton-Carafa Y, Xu ZY, Steinmetz LM, Jacquier A. Widespread bidirectional promoters are the major source of cryptic transcripts in yeast. Nature. 2009;457: 1038–1042. 10.1038/nature07747 19169244

[52] Qutob D, Patrick Chapman B, Gijzen M. Transgenerational gene silencing causes gain of virulence in a plant pathogen. Nature Comm. 2013;4: 1349. 10.1038/ncomms2354 PMC3562452 23322037

[53] Kim JH, Ebersole T, Kouprina N, Noskov VN, Ohzeki JI, Masumoto H, et al Human gamma-satellite DNA maintains open chromatin structure and protects a transgene from epigenetic silencing. Genome Res. 2009;19:533–544. 10.1101/gr.086496.108 19141594 PMC2665773

[54] Hodges BL, Taylor KM, Joseph MF, Bourgeois SA, Scheule RK. Long-term transgene expression from plasmid DNA gene therapy vectors is negatively affected by CpG dinucleotides. Mol Ther. 2004;10:269–278. 15294174 10.1016/j.ymthe.2004.04.018

[55] Kishimoto N, Nagai J, Kinoshita T, Ueno K, Ohashi Y, Mitsuhara I. DNA elements reducing transcriptional gene silencing revealed by a novel screening strategy. PloS One. 2013;8: e54670 10.1371/journal.pone.0054670 23382937 PMC3559876

