# Supplementary material for: Comparisons of Ribosomal Protein Gene Promoters Indicate Superiority of Heterologous Regulatory Sequences for Expressing Transgenes in Phytophthora infestans
Source: PLoS One. 2015 Dec 30;10(12):e0145612. doi: 10.1371/journal.pone.0145612 (PMC4696810; doi:10.1371/journal.pone.0145612)

S1 Figure

Distribution of introns in genes encoding ribosomal proteins (red line) and in 200 random intron-containing genes (blue line). Each bin represents the distance from the start codon to the nearest intron. The bin labelled <10 includes some introns that are upstream of the start codon.

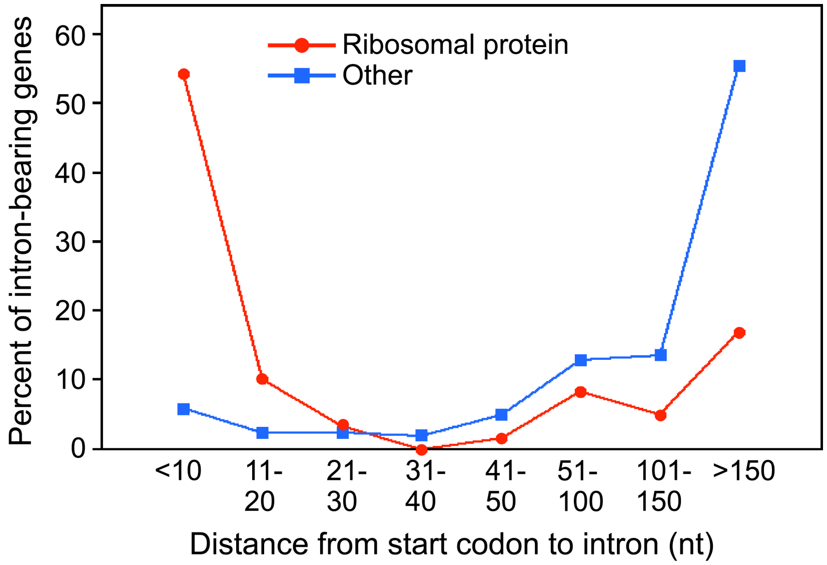

Supplement: S1 Fig — (PDF) [file pone.0145612.s001.pdf]
