# Supplementary material for: Comparisons of Ribosomal Protein Gene Promoters Indicate Superiority of Heterologous Regulatory Sequences for Expressing Transgenes in Phytophthora infestans
Source: PLoS One. 2015 Dec 30;10(12):e0145612. doi: 10.1371/journal.pone.0145612 (PMC4696810; doi:10.1371/journal.pone.0145612)
Supplement: S2 Fig — (PDF) [file pone.0145612.s002.pdf]

S2 Figure

Letter-probability matrix for PhRiboBox, determined using MEME with ribosomal promoter dataset from *P. infestans.*

0.366197	0.140845	0.394366	0.098592
0.577465	0.056338	0.267606	0.098592
0.295775	0.084507	0.140845	0.478873
0.000000	0.267606	0.000000	0.732394
0.000000	0.028169	0.971831	0.000000
0.535211	0.000000	0.464789	0.000000
0.084507	0.366197	0.000000	0.549296
0.000000	0.000000	0.000000	1.000000
0.000000	0.000000	1.000000	0.000000
0.000000	0.000000	1.000000	0.000000
0.000000	0.633803	0.014085	0.352113
0.056338	0.197183	0.253521	0.492958
0.126761	0.366197	0.450704	0.056338
0.225352	0.183099	0.295775	0.295775
0.211268	0.352113	0.281690	0.154930
0.366197	0.126761	0.450704	0.056338
