# Supplementary material for: Comparisons of Ribosomal Protein Gene Promoters Indicate Superiority of Heterologous Regulatory Sequences for Expressing Transgenes in Phytophthora infestans
Source: PLoS One. 2015 Dec 30;10(12):e0145612. doi: 10.1371/journal.pone.0145612 (PMC4696810; doi:10.1371/journal.pone.0145612)
Supplement: S3 Fig — (PDF) [file pone.0145612.s003.pdf]

S3 Figure

Alignment of promoter regions of *RPS9* orthologs from (top to bottom) *P. infestans, P. parasitica, P. capsici,* and *P. ramorum.* Sequences shown extend 325-nt upstream of the start codon. The location of the strongest PhRiboBox is boxed, and CCAAT motifs (in minus orientation compared to Fig. 1B) are shaded in grey. The transcription start site in the *P. infestans* gene is just downstream of the PhRiboBox, based on the analysis of RNA-seq data. This is not the case in other ribosomal protein genes from *P. infestans,* however.

```
PINFS9   TTGTCGCACCGGCTTATGTAATGAAGTGAATTGGTATGCC--AAAATCGGTTGCATGTAA 58
PPARS9   --TTTGATTTGATCTTTGTAA---GGTGA--TAGTG------AATGTCGCTTGTGGGTAA 47
PCAPS9   --------ATGGC-TGTGAAATCGGTCGCTTTGCTCCCCCTCAAAGTCGAAAGTCAGGAG 51
PRAMS9   ----------CATGCTTGAAA---GTCAA--TGGTGGCCAGGGGGGCGAGCCTCCCGCCA 45
                         ** **          *  *                     *

PINFS9   ----CGTACCTATATG-TACCAGTTCCAGATAGAAAT-ATTCTAAAC--TCGTAACTCCC 110
PPARS9   ATGTTGCACCAAAAGA-GAAT-GTT---GGTAGATTG-ATTTCAGCCAATCGTAGCTAC- 100
PCAPS9   -------AGCCACA----AACGGTT---GGTAGAAGCCACTGCTGCAA--CATTCCTTCC 95
PRAMS9   GCCGCACATTTTCAGCCTATCCGGT---GATGGATACAA-----------CATT-CTTC- 89
                *     *    *   * *   * * **    *           * *  ** *

PINFS9   GGGTAGGACTCTGGTGGTGGGCCGGAA---GGTTTTCCCGAGA----AAACCCG--ATAT 161
PPARS9   -ATTA----TCCGTCTATTGGCTGTAA---AAAGTT-----AA----GTACTAA--GTCT 141
PCAPS9   AATTGTTGCTCGTCGTGTAAGCCACATC--AGCTTTCTC--AA----CTCCCCC--TTTT 145
PRAMS9   ---TG----CACGCAGACTGGCTGAAAACAAAACCTCCCAGAATTGTGTATTTATCATTT 142
            *                **   *         *      *              * *

PINFS9   ---ACCGGTAATTATTCTACAGG--AAGGACCGGTTCTAACCGGT----AATCCACCT-- 210
PPARS9   C--ACAGGAAATAATATCACTTCTCACAGTCTCCTTCTGACTGGTTGAAAATGTCCTTTT 199
PCAPS9   TTGATTCGTGAAAACAATACAATTCTATGATTGACTGAAAATCA-----AACGCCCTTT- 199
PRAMS9   CCCACAGGTATATATATTGATTCTTACAGGTCAGTGTTCATTGGTC-AAAATGAGATCGT 201
            *   *     *              *          *         **
                                   CCAAT box

PINFS9   --CGCGACATGATTGGCGGATGAGT-ATTGGTGGTTGGCTGG-TGCAGGAGC-------- 258
PPARS9   TCCGCGATCTGATTGGCTGATGAAT-ATTGGTGGTTGGCTGG-TGCAGAAGC-------- 249
PCAPS9   -CAGCCA-ATCATAGAACGTTGGCTGATTGGTGGTTGGCTGG-TGCAGACGCGCTAGCTG 256
PRAMS9   GCCGGGCCGCGATTGGCTGACAGAT-ATTGGTGGTTGGCTGGCTGCAGGCGG-------- 252
            *       ** *   *     * **************** *****  * P. infestans
                                  PhRiboBox        transcription start

PINFS9   -TCTTGGCGCATT----------AGGCCCCGACGGCTCCCACTCCCATCGCATTCGCCTG 307
PPARS9   -TTTTGGCGCATTGTGGTCTTGCAGCCCCCGACGTCTCC-ACTCCCATCGCATTCGCCTG 307
PCAPS9   CTCTTGTTGCTTT----------CGCCCCCAGCGTCCGCCACTCCCATCGCATTCGCCTG 306
PRAMS9   CGCTCGCCATGCCCGCGACG---TGCCCCCGACGTCTCC-ACTCCCATCGCATTCGCCTG 308
            * *                  * ****  ** *  * ********************

PINFS9   AA--AAAGAAGTCAACCAACATG 328
PPARS9   AA--AAAGAAGTCACGAAAAATG 328
PCAPS9   AACAAAAGAAGTCG-CCAAAATG 328
PRAMS9   AA--AAAGAAGTCT-CGAAAATG 328
         **  *********    ** ***
                         Start codon
```
