# Supplementary material for: Comparisons of Ribosomal Protein Gene Promoters Indicate Superiority of Heterologous Regulatory Sequences for Expressing Transgenes in Phytophthora infestans
Source: PLoS One. 2015 Dec 30;10(12):e0145612. doi: 10.1371/journal.pone.0145612 (PMC4696810; doi:10.1371/journal.pone.0145612)

S4 Figure

Relative expression of PiRPL10, PiRPL23, and PiRPS9 in rye broth (3 day non-sporulating cultures) and in tomato leaflets at 3 and 6 days post-infection. Sporulation had occurred in the plant samples on day 5.

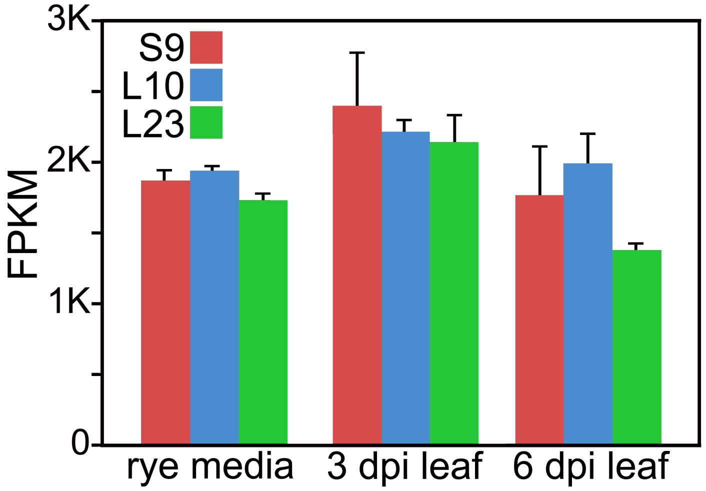

Supplement: S4 Fig — (PDF) [file pone.0145612.s004.pdf]
