# Supplementary material for: Comparisons of Ribosomal Protein Gene Promoters Indicate Superiority of Heterologous Regulatory Sequences for Expressing Transgenes in Phytophthora infestans
Source: PLoS One. 2015 Dec 30;10(12):e0145612. doi: 10.1371/journal.pone.0145612 (PMC4696810; doi:10.1371/journal.pone.0145612)
Supplement: S1 Table — (PDF) [file pone.0145612.s005.pdf]

S1 Table. Primers used for polymerase chain reaction.

Name (prefix is gene name)	Forward and reverse primers (5' to 3')
PITG_19121-500	TCTAGAGAAAGTGCTCCATTCAGGGCAGGGGT, GAATTCCTTAGACTTATTTGGCA AATTCCACA
PITG_01943-500	TCTAGATGCGCAGCCTCCTCAATCTGCTTCA, GAATTCTGTGCTGCTTCCTTTAGAGAATTAC
PITG_09563-500	TCTAGACTACTATGGAGGATGGCTTTTAGAGGGTG, GAATTCGTTGGTTGACTTCTTTTTCAGGC
PC_93109-500	TCTAGATTAGCCATATCTAGCTCATATTTGGC, GAATTCTGTTGCTGCTTCCTTTAGAGAATTAC
PC_91078-500	TCTAGATATTTTCAACGTCAGCGGCTCTTTACA, GAATTCTGTGACTTTTTCTTGGCAAATTGCTC
PC_89970-500	TCTAGACGTCAATACGGCTGTAAACCACAGGAC, GAATTCTTTGGCGACTTCTTTTGTTCAGGCG
PC_89970-500-TOR	TCTAGACGTCAATACGGCTGTAAACCACAGGAC, CTGAATTCGATATCTTTGGCGACTTCTTTTGT
PC_89970-420-TOR	TTATACGTAGACCGCCACCTTGGCCA, CTGAATTCGATATCTTTGGCGACTTCTTTTGT
PC_89970-325-TOR	CTATACGTACGTCAATACGGCTGTAAACCACAGGAC, CTGAATTCGATATCTTTGGCGACTTCTTTTGT
PiRPL10	TTATCTCGGTGCGTTCCAAG, CATCTCAGCGTACACCTCAC
PiRPS9	CGAAGATTTCGCGCCACC, GTACTGCACACGCCAGATC
PITG_11766	TAAGACGACGGACGGATAC, AGGCACAAACTTCAGGAATAG
