# Supplementary material for: Comparisons of Ribosomal Protein Gene Promoters Indicate Superiority of Heterologous Regulatory Sequences for Expressing Transgenes in Phytophthora infestans
Source: PLoS One. 2015 Dec 30;10(12):e0145612. doi: 10.1371/journal.pone.0145612 (PMC4696810; doi:10.1371/journal.pone.0145612)
Supplement: S2 Table — (PDF) [file pone.0145612.s006.pdf]

S2 TABLE **NUMBER OF GENES ENCODING RIBOSOMAL PROTEINS FROM SELECTED EUKARYOTES**

			number of genes													
			oomycete			diatom		fungus		plant		animal		Features of *P. infestans* gene		
Subunit	Protein name	Corresponding gene in *P. infestans*	*Phytophthora infestans*	*Phytophthora capsici*	*Phytophthora parasitica*	*Thalassiosira pseudonana*	*Phaeodactylum tricornutum*	*Neurospora crassa*	*Schizosaccharo myces pombe*	*Arabidopsis thaliana*	*Glycine max*	*Homo sapiens*	*Drospophila melanogaster*	5' intron present?	PhRiboBox present within 200 nt of ATG?	CCAAT motif present within 200 nt of ATG?
Small (40S)	SA	PITG_01922	1	1	1	1	1	1	2	2	4	1	1	N	Y	N
	S2	PITG_10146	1	1	1	1	1	1	1	4	3	1	1	N	N	Y
	S3	PITG_08959	1	1	1	1	1	1	1	3	6	1	1	N	Y	Y
	S3A	PITG_11766	1	1	1	1	1	1	2	2	5	1	1	Y	N	N
	S4	PITG_13500	1	1	1	1	1	1	2	3	6	2	1	N	Y	Y
	S5	PITG_09345	1	1	1	1	1	1	1	2	4	1	2	Y	N	N
	S6	PITG_00443	1	1	1	1	1	1	2	2	7	1	1	Y	Y	N
	S7	PITG_14850	1	1	1	1	1	1	2	3	6	1	1	N	N	N
	S8	PITG_02053	1	1	1	1	1	1	2	2	5	1	1	Y	Y	Y
	S9	PITG_09563	1	1	1	1	1	1	2	2	4	1	1	N	Y	Y
	S10	PITG_00266	1	1	1	1	1	1	2	3	4	1	2	N	Y	Y
	S11	PITG_10887	1	2	1	1	1	1	2	3	4	1	1	Y	Y	Y
	S12	PITG_00179	1	1	1	1	1	1	1	2	4	1	1	N	Y	N
	S13	PITG_20264	1	1	1	1	1	1	1	2	3	1	1	N	N	Y
	S14	PITG_19999	1	1	1	1	1	1	2	3	6	1	2	Y	Y	Y
	S15	PITG_07173	1	1	1	1	1	1	1	5	3	1	1	N	Y	N
	S15A	PITG_16757	1	1	1	1	1	1	2	4	7	1	2	Y	Y	Y
	S16	PITG_14913	1	1	1	1	1	1	2	3	4	1	1	N	N	N
	S17	PITG_12745	1	1	1	1	1	1	2	4	4	1	1	N	Y	N
	S18	PITG_03221	1	1	1	1	1	1	2	3	4	1	1	Y	Y	Y
	S19	PITG_09431	1	1	1	1	1	1	2	3	4	1	2	Y	Y	Y
	S20	PITG_12299.2	1	1	1	1	2	1	1	3	5	1	1	N	Y	Y
	S21	PITG_09442	1	1	1	1	1	1	2	2	3	1	1	Y	N	N
	S23	PITG_12947	1	1	1	1	1	1	2	2	4	1	1	Y	N	Y
	S24	PITG_06771	1	1	1	1	1	1	2	2	4	1	1	Y	Y	Y
	S25	PITG_13312	1	1	1	1	1	1	2	2	4	1	1	Y	N	N
	S26	PITG_13681	1	1	1	1	1	1	2	3	5	1	1	N	Y	Y
	S27	PITG_16008	1	1	1	1	1	1	2	3	5	1	1	N	N	N
	S27A	PITG_10193	1	1	1	1	1	1	1	3	5	1	1	Y	N	Y
	S28	SC237: 71896-72030 -	1	1	1	1	1	1	2	3	4	1	2	Y	N	N
	S29	PITG_00631	1	1	1	1	2	1	2	3	4	1	1	Y	N	N
	S30	PITG_19669	1	1	1	1	1	2	2	3	3	1	1	Y	Y	N
Large (60S)	L10	PITG_19121	1	1	1	1	1	1	1	2	5	1	1	N	N	Y
	L10A	PITG_22135	1	1	1	1	1	1	2	2	5	1	2	N	Y	N
	L11	PITG_15697	1	1	1	1	1	1	2	4	6	1	1	Y	Y	Y
	L12	PITG_08809	1	1	1	1	1	1	2	3	4	1	1	N	N	N
	L13	PITG_04843	1	1	1	1	1	1	2	3	3	1	1	Y	N	Y
	L13A	PITG_01833	1	1	1	1	1	1	2	4	4	1	1	Y	Y	Y
	L14	PITG_15723, PITG_20189	2	1	1	1	1	1	2	2	4	1	1	Y, Y	Y, Y	Y, Y
	L15	PITG_02578	1	1	1	1	1	1	2	2	4	1	1	N	Y	N
	L17	PITG_03353	1	1	1	1	1	1	2	2	4	1	1	N	Y	Y
	L18	PITG_11099	1	1	1	1	1	1	2	2	6	1	1	Y	Y	N
	L18A	PITG_16198	1	1	1	1	1	1	2	3	5	1	1	Y	N	Y
	L19	PITG_09540	1	1	1	1	1	1	2	3	8	1	1	Y	Y	N

L21	PITG_00941	1	1	1	1	1	1	2	3	2	1	1	N	Y	Y
L22	PITG_01042	1	1	1	1	1	1	2	3	5	1	1	Y	Y	Y
L23	PITG_01943	1	1	1	1	1	1	2	3	4	1	1	Y	Y	N
L23A	PITG_02694	1	1	1	1	1	1	1	2	6	1	1	N	N	N
L24	PITG_17093	1	1	1	1	1	1	2	2	4	1	1	Y	Y	Y
L26	SC1:2501105-2501723 +	1	1	1	1	1	1	2	2	5	1	1	N	Y	N
L27	PITG_03239	1	1	1	1	1	1	2	3	4	1	1	Y	Y	N
L27A	PITG_06799	1	1	1	1	1	1	1	2	6	1	1	Y	Y	Y
L28	PITG_03916	1	1	1	1	1	1	0	2	4	1	1	Y	Y	Y
L29	PITG_10202	1	1	1	1	1	1	1	2	4	1	1	N	Y	N
L3	PITG_18052	1	1	1	1	1	1	1	2	4	1	1	N	Y	Y
L30	PITG_00879	1	1	1	1	1	1	1	3	3	1	1	Y	Y	Y
L31	PITG_13371	1	1	1	1	1	1	2	3	4	1	1	Y	Y	N
L32	PITG_10863	1	1	1	1	1	1	1	2	7	1	1	Y	Y	N
L34	PITG_15407	1	1	1	1	1	1	2	3	6	1	2	Y	Y	N
L35	PITG_07300	1	1	1	1	1	1	2	4	5	1	1	Y	Y	Y
L35A	PITG_03235	1	1	1	1	1	1	2	4	4	1	1	Y	Y	Y
L36	PITG_08703	1	1	1	1	1	1	2	3	3	1	1	Y	Y	N
L36A	PITG_05171	1	1	1	1	2	1	2	2	4	1	1	Y	Y	Y
L37	PITG_08834	1	1	1	1	1	1	2	3	4	1	1	Y	Y	Y
L37A	PITG_10263	1	1	1	1	1	1	2	2	6	1	1	Y	Y	Y
L38	PITG_03762	1	1	1	1	1	1	1	2	4	1	1	Y	N	Y
L39	PITG_13676	1	1	1	1	1	1	1	2	5	1	1	Y	Y	Y
L4	PITG_06995	1	1	1	1	1	1	2	2	4	1	1	N	Y	N
L40	PITG_09552, PITG_09555	2*	1	1	2	1	1	2	2	4	1	1	N, Y	N,N	N,N
L41	PITG_15069	1	1	1	1	1	1	2	4	0	1	1	Y	N	N
L5	PITG_20188, PITG_15722	2	1	1	1	1	1	1	2	5	1	1	N, N	Y	Y
L6	PITG_03294, PITG_03660	2*	2*	2*	1	1	1	2	3	6	1	1	Y, Y	Y, N	N,N
L7	PITG_07269	1	1	1	1	1	1	2	3	6	1	1	Y	Y	Y
L7A	PITG_03477	1	1	1	1	1	1	2	2	4	1	1	Y	Y	Y
L8	PITG_06237	1	1	1	1	1	1	2	3	4	1	1	N	Y	Y
L9	PITG_03178	1	1	1	1	1	1	2	3	4	1	1	N	N	Y
LP0	PITG_17261	1	1	1	1	1	1	1	3	3	1	1	N	Y	Y
LP1	PITG_03486, PITG_15638	2	2	2	2	1	1	2	3	4	1	1	N	Y,N	N,N
LP2	PITG_01041, PITG_20795	2	2	2	1	2	1	1	5	6	1	1	Y, Y	Y, Y	Y, Y
LP3		0	0	0	0	0	0	0	2	0	0	0			
TOTAL NUMBER OF GENES		85	83	82	81	83	80	136	217	354	80	87			

* one is a likely pseudogene.
