# Supplementary material for: Comparisons of Ribosomal Protein Gene Promoters Indicate Superiority of Heterologous Regulatory Sequences for Expressing Transgenes in Phytophthora infestans
Source: PLoS One. 2015 Dec 30;10(12):e0145612. doi: 10.1371/journal.pone.0145612 (PMC4696810; doi:10.1371/journal.pone.0145612)
Supplement: S3 Table — (PDF) [file pone.0145612.s007.pdf]

S3 Table

P. infestans genes containing the PhRiboBox in the 200-nt region upstream of their start codons, that contain the GO annotation for Nucleic Acid Binding (GO:0003676).

Gene ID	Product description from Fungidb or Panther
PITG_19178	13 kDa ribonucleoprotein-associated protein
PITG_03221	40S ribosomal protein S18
PITG_08959	40S ribosomal protein S3-3
PITG_13500	40S ribosomal protein S4
PITG_09563	40S ribosomal protein S9-1
PITG_22135	60S ribosomal protein L10a-1
PITG_20188	60S ribosomal protein L5
PITG_06237	60S ribosomal protein L8
PITG_12151	Aspartyl-tRNA synthetase
PITG_00499	ATP-dependent RNA helicase DBP4
PITG_13017	ATP-dependent RNA helicase
PITG_19163	ATP-dependent RNA helicase
PITG_01246	Signal recognition particle protein
PITG_01361	CCR4-NOT transcription complex subunit 4
PITG_01682	Nucleolar protein 8
PITG_02480	C2H2 transcription factor
PITG_02716	Centromere protein b
PITG_02889	Nucleolar protein 6
PITG_03308	Pumilio RNA binding protein
PITG_03939	Telomere binding protein SDE2
PITG_03956	Eukaryotic translation initiation factor 3 subunit k
PITG_04334	Ribosomal L1 domain-containing protein 1
PITG_04567	small nuclear ribonucleoprotein assembly protein SMN
PITG_04664	RING-type zinc finger protein
PITG_04956	CCHC ZF RNA binding protein
PITG_06416	Transcription factor E2F
PITG_06706	DNA-directed RNA polymerase 1subunit RPA12
PITG_07888	Translation initiation factor IF-3, mitochondrial
PITG_08410	Myb-like DNA-binding protein
PITG_08709	Pin2/terf1-interacting telomerase inhibitor
PITG_08932	tRNA selenocysteine 1-associated protein
PITG_12100	PHD finger transcription factor
PITG_12426	Myb-like protein G
PITG_14134	Serine/arginine-rich splicing factor 7
PITG_15740	Methyltransferase NSUN6-like
PITG_16548	RNA methyltransferase
PITG_17089	Transcription initiation factor TFIID subunit 13
PITG_17706	cyclic AMP-response element binding protein

PITG_17733	Signal recognition particle 14kd protein
PITG_18056	CCCH RNA-binding protein
PITG_18100	Lupus la protein-related ribonucleoprotein
PITG_19007	39S ribosomal protein L21, mitochondrial
PITG_19968	G-patch RNA processing protein
PITG_20209	MYB-like protein
PITG_00227	DEAD/DEAH box RNA helicase
PITG_02839	DEAD/DEAH box RNA helicase
PITG_02856	DEAD/DEAH box RNA helicase
PITG_03304	DEAD/DEAH box RNA helicase
PITG_06827	DEAD/DEAH box RNA helicase
PITG_06981	DEAD/DEAH box RNA helicase
PITG_09792	DEAD/DEAH box RNA helicase
PITG_12165	DEAD/DEAH box RNA helicase
PITG_21398	DEAD/DEAH box RNA helicase
PITG_15505	DNA polymerase epsilon catalytic subunit
PITG_03713	DNA polymerase kappa
PITG_03516	DNA polymerase lambda-like protein
PITG_08956	DNA repair protein REV1
PITG_13457	DNA repair protein XRCC3
PITG_07399	DNA replication licensing factor MCM5
PITG_11912	DNA replication licensing factor MCM7
PITG_18777	DNA-directed RNA polymerase I and III subunit RPAC1
PITG_03855	DNA-directed RNA polymerase I subunit RPA1
PITG_02420	DNA-directed RNA polymerase I subunit RPA2
PITG_10445	DNA-directed RNA polymerase I, II, and III subunit RPABC1
PITG_18113	DNA-directed RNA polymerase II 36 kDa polypeptide A
PITG_00051	DNA-directed RNA polymerase III subunit RPC6
PITG_03262	double stranded RNA binding protein
PITG_10974	Elongation factor 1-gamma
PITG_12524	Elongation factor 3
PITG_17205	Elongation factor G
PITG_03274	Elongation factor P
PITG_06048	Eukaryotic initiation factor 4a-III
PITG_03999	Eukaryotic initiation factor 4e
PITG_02493	Eukaryotic translation initiation factor 1b
PITG_06440	Eukaryotic translation initiation factor 2a
PITG_00397	Eukaryotic translation initiation factor 3 subunit c
PITG_17240	Euk. translation initiation factor 3 subunit e-interacting protein
PITG_06482	Eukaryotic translation initiation factor 3 subunit
PITG_12483	Eukaryotic translation initiation factor 3
PITG_16834	Exodeoxyribonuclease
PITG_15606	Exosome complex exonuclease RRP41-like protein

PITG_18509	Exosome complex exonuclease RRP44
PITG_07327	H/aca ribonucleoprotein complex non-core subunit NAF1
PITG_05391	Histone H1
PITG_02527	Histone H2a
PITG_03611	Histone H2b
PITG_06950	Histone H3.3
PITG_05675	Histone H4
PITG_08199	Hsf-type DNA-binding
PITG_07234	Ligatin
PITG_06978	Metalloprotease family M20D
PITG_00988	Myb-like dna-binding protein
PITG_08960	Myb-like dna-binding protein
PITG_19362	Myb-like dna-binding protein
PITG_01756	Negative elongation factor
PITG_10861	Nuclear transcription factor Y subunit B-3
PITG_02629	Nucleolin
PITG_08368	Peptidyl-prolyl cis-trans isomerase-like protein
PITG_03778	Poly(a) polymerase
PITG_01248	Polyadenylate-binding protein 1-B
PITG_14397	Proliferating cell nuclear antigen
PITG_13792	Ribonuclease H2 subunit a
PITG_12152	Ribonuclease H2
PITG_16015	Ribonuclease
PITG_08834	Ribosomal protein L37e
PITG_19608	Ribosomal protein L7ae/L30e/S12e/gadd4
PITG_02450	rRNA assembly protein MIS3
PITG_02752	rRNA methyltransferase NOP2-like protein
PITG_10316	RNA exonuclease 4
PITG_13393	RNA-binding protein
PITG_07308	rRNA 2'-o-methyltransferase fibrillarin
PITG_16107	rRNA biogenesis protein RRP5
PITG_00663	rRNA methyltransferase
PITG_04538	Splicing factor 3 subunit
PITG_03791	Splicing factor, arginine/serine-rich
PITG_16524	Spou rRNA methylase family
PITG_18518	Translation initiation factor eif-2b subunit alpha
PITG_01925	Transmembrane protein
PITG_09866	tRNA (adenine-N(1)-)-methyltransferase subunit TRM6
PITG_17298	tRNA (cytosine-5-)-methyltransferase
PITG_20377	tRNA pseudouridine synthase
PITG_09987	tRNA pseudouridine synthase
PITG_06262	U3 small nucleolar ribonucleoprotein protein IMP3
PITG_02211	UV excision repair protein RAD23
